# RIVER Technique: Retrograde IVUS Endovascular Revascularization of Anomalous Tibial Chronic Total Occlusions

**DOI:** 10.1016/j.jaccas.2025.106601

**Published:** 2026-01-21

**Authors:** Shadi Halabi, Eric Gonsiorowski, Wael Gad, Fares Chamma, Andrej Schmidt

**Affiliations:** a Department of Cardiology, Community Hospital, Powers Health, Munster, Indiana, USA; b Indiana University School of Medicine, Indianapolis, Indiana, USA; c Division of Angiology, University Hospital Leipzig, Leipzig, Germany

**Keywords:** intravascular ultrasound, peripheral vascular disease, treatment

## Abstract

**Case Summary:**

A 62-year-old man presented with a long-standing history of a nonhealing ulcer to the left foot. Previous antegrade revascularization attempts failed. Retrograde access was obtained at the posterior tibial artery, but the wire traveled away from the tibial peroneal trunk and connected at the popliteal artery. Intravascular ultrasound confirmed intraluminal passage of the wire, which led to successful revascularization of the posterior tibial artery and ulcer healing.

**Take-Home-Message:**

The RIVER (Retrograde Intravascular ultrasound Endovascular Revascularization of Anomalous Tibial Chronic Total Occlusions) technique is a useful strategy for revascularization of anomalous tibial occlusions when standard antegrade approaches fail.

Chronic limb-threatening ischemia is a serious manifestation of peripheral artery disease and is associated with poor outcomes including longer hospital stays, increased amputation rate, and higher mortality.^1^ Successful revascularization of tibial chronic total occlusions can be complicated by anatomic variants. Revascularization of these anomalous vessels has not been well described in the literature despite variation in tibial literature being found in up to 10% of patients.^2^ This case highlights the RIVER (Retrograde Intravascular ultrasound Endovascular Revascularization of Anomalous Tibial Chronic Total Occlusions) technique where a combination of retrograde access and intravascular ultrasound (IVUS) can aid in the successful and safe revascularization of such anatomic variants.

A 62-year-old man with a long-standing nonhealing ulcer to the left foot presented for a second opinion after failing multiple revascularizations attempts and being recommended a below-knee amputation. To further evaluate he was scheduled for an angiogram. Antegrade access was obtained at the common femoral artery. The posterior tibial artery (PTA) was occluded with no clear take-off (Figure 1A). Attempts were made at wiring the PTA near the tibial peroneal trunk expected take-off but were unsuccessful. Identification of the PTA ostium via IVUS guidance was attempted, but once again the PTA opening was not found.^3^ Next, retrograde access was obtained at the distal PTA. The PTA was calcified and underperfused, requiring multiple ultrasound-guided attempts for canalization. The wire was advanced up the left PTA where it was noted to take a path away from the typical tibial peroneal trunk. The wire from the PTA connected to the proximal popliteal artery (Figure 1A). Before performing balloon angioplasty, IVUS was performed, confirming an intraluminal wire crossing of the calcified PTA (Figure 1C). Successful balloon angioplasty was performed on the PTA restoring in-line flow to the wound (Figures 1A and 1B). The patient experienced wound healing with continued care at the wound clinic with prevention of a major amputation.

**Figure 1:**
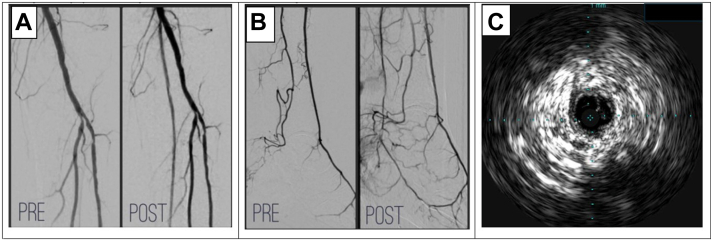
RIVER Technique for Revascularization of an Anomalous Posterior Tibial Artery (A) Pre- and postrevascularization of anomalous posterior tibial artery. (B) Pre- and postrevascularization of dorsal pedal loop. (C) Intravascular ultrasound image showing intraluminal access of anomalous posterior tibial artery.

The anomalous nature of an artery provides a unique challenge to successful revascularization by adding another layer of complexity including finding the vessel origin and ensuring true lumen access of the wire. We present a case of a patient with a nonhealing dorsal foot ulcer where we were unable to find the PTA take-off from the tibial peroneal trunk. The combination of retrograde access and IVUS proved to be essential in safely revascularizing the diseased anomalous artery.

## Funding Support and Author Disclosures

The authors have reported that they have no relationships relevant to the contents of this paper to disclose.
